# Systematic analysis of healthcare big data analytics for efficient care and disease diagnosing

**DOI:** 10.1038/s41598-022-26090-5

**Published:** 2022-12-26

**Authors:** Sulaiman Khan, Habib Ullah Khan, Shah Nazir

**Affiliations:** 1 Department of Accounting and Information Systems, College of Business and Economics, Qatar University, Doha, Qatar; 2 Department of Computer Science, University of Swabi, Swabi, Pakistan

**Keywords:** Biotechnology, Computational biology and bioinformatics

## Abstract

Big data has revolutionized the world by providing tremendous opportunities for a variety of applications. It contains a gigantic amount of data, especially a plethora of data types that has been significantly useful in diverse research domains. In healthcare domain, the researchers use computational devices to extract enriched relevant information from this data and develop smart applications to solve real-life problems in a timely fashion. Electronic health (eHealth) and mobile health (mHealth) facilities alongwith the availability of new computational models have enabled the doctors and researchers to extract relevant information and visualize the healthcare big data in a new spectrum. Digital transformation of healthcare systems by using of information system, medical technology, handheld and smart wearable devices has posed many challenges to researchers and caretakers in the form of storage, minimizing treatment cost, and processing time (to extract enriched information, and minimize error rates to make optimum decisions). In this research work, the existing literature is analysed and assessed, to identify gaps that result in affecting the overall performance of the available healthcare applications. Also, it aims to suggest enhanced solutions to address these gaps. In this comprehensive systematic research work, the existing literature reported during 2011 to 2021, is thoroughly analysed for identifying the efforts made to facilitate the doctors and practitioners for diagnosing diseases using healthcare big data analytics. A set of rresearch questions are formulated to analyse the relevant articles for identifying the key features and optimum management solutions, and laterally use these analyses to achieve effective outcomes. The results of this systematic mapping conclude that despite of hard efforts made in the domains of healthcare big data analytics, the newer hybrid machine learning based systems and cloud computing-based models should be adapted to reduce treatment cost, simulation time and achieve improved quality of care. This systematic mapping will also result in enhancing the capabilities of doctors, practitioners, researchers, and policymakers to use this study as evidence for future research.

## Introduction

Healthcare around the world is under high pressure due to limiting financial resources, over-population, and disease burden. In this modern technological age the healthcare paradigm is shifting from traditional, one-size-fits-all approach to a focus on personalized individual care^1^. Additionally, the healthcare data is varying both in type and amount. The healthcare providers are not only dealing with patient’s historical, physical and namely information, but they also deal with imaging information, labs, and other digital and analogue information consists of ECG, MRI etc. This data is voluminous, varying in type and formats, and of differing structure. These are the capabilities of Big Data to handle not only different types of and forms of data, but can handle 10 V structure including volume, variety, venue, varifocal, varmint, vocabulary, validity, volatility, veracity and velocity. Thus, the doctors facing an increasing burden of rising patient numbers coupled with progressively less time to spend with each patient. In other words, we are facing more patients, more data, and less time.

Big data has significantly attracted the researchers to explore different research fields including healthcare, banking, imaging, smart cities, internet of things (IoT) based smart applications, tracking and transportation system etc.^2^. Software engineers constantly develops new applications for patient’s health and well-being. Both government and non-government organizations develop infrastructure using big data analytics for improved decision making capabilities of both doctors and managers^3^. It was recorded that 80% increase in big data is due to cloud sources, big data analytics, mobile technology and social media technologies^4^. A number of research articles proposed using big data analytics in varying domains especially in healthcare such as Kumar et al.^5^ proposed a cognitive technology-based healthcare evaluations system using big data analytics. Chen et al.^6^ presented an intelligent healthcare application for brain hemorrhage detection using Big Data analytics and machine learning (ML) techniques. Smart health appointment system is developed by Liang and Zhao using big data analytics is^7^.

Some researchers explored big data analytics in healthcare domain in different ways. They presented survey papers and review papers to understand the meanings of big data analytics in healthcare such as Galetsi and Kasaliasi performed a review of healthcare big data analytics^8^ while Lindell defined big data analytics in terms of accounting and business perspectives^9^. Alharthi proposed a review article on healthcare challenges facing in Saudi Arabia by performing analysis of the available literature^10^. Lee et al.^11^ presented a survey paper to explore the applications and challenges of healthcare big data analytics. From the literature it is concluded that multiple new applications are developed for big data analysis. Review and survey papers are presented to outline the published literature, but most of these papers are region specific or limited to a few numbers of papers. On the other side systematic review process formulate multiple research question and identifies keywords to explore the available literature from different angles. Systematic analysis of the available literature is presented in many fields like PMIPv6 domain^12^, in smart homes^13^, navigation assistants^14^, and many others, but there is no significant work reported on systematic analysis for healthcare big data domain to find the gaps in the available literature and suggest future research directions.

The inspirational point that led us to pursue this systematic analysis was the pervasive and ubiquitous nature of big data. Efficient management and timely execution are the dire needs of big data, to extract enriched information regarding a certain problem of interest^15^. Many factors involved behind this systematic research work, but the most eminent reasons are:

i. The exiting research reported on big data does not provide significant information about the key features that should be considered to integrate both structured and unstructured big data in healthcare domain. The pervasiveness of big data features challenging the researchers in pursuing research in this specialized domain. The underlying research on finding the key features will not only help in integrating big data in healthcare domain, but it will also assist in findings new gateways for future research directions.
ii. Digital transformation of healthcare systems after the integration of information system, medical technology and other imaging systems have posed a big barrier for the research community in the form of a vast amount of information to deal with. While the over-population, limited data access, and disease burdens have restricted the doctors and practitioners to check more patients in a limited time. So, finding a suitable model that can efficiently process healthcare big data to extract information for a certain disease symptoms will not only helps the practitioners to suggest accurate medication and check more patients in timely manners, but it will open future research directions for the industrialists and policymakers to develop optimal healthcare big data processing models.
iii. Accurate disease diagnosing by processing of gigantic amount of data, especially a plethora of types of data, within an interested processing domain is a key concern for both researchers and practitioners. Developing an efficient model that can accurately diagnose a certain by classifying images or other historical details of patients will not only helps the doctors to diagnose disease in timely manner and suggest medicine accordingly, but it will encourage the researchers and developers to develop an accurate disease identification model.

The remaining research paper of the paper is organized as follows. Section 2 of the paper outlines the related work reported in the proposed field. Section 3 presents the research framework followed for this systematic research work. Quality assessment is detailed in Sect. 4. Section 5 outlines the discussion on findings of the proposed systematic research work. Section 6 provides the limitations of this systematic study traced by the conclusion and future work in Sect. 7 of the paper.

## Literature review

**Figure 1:**
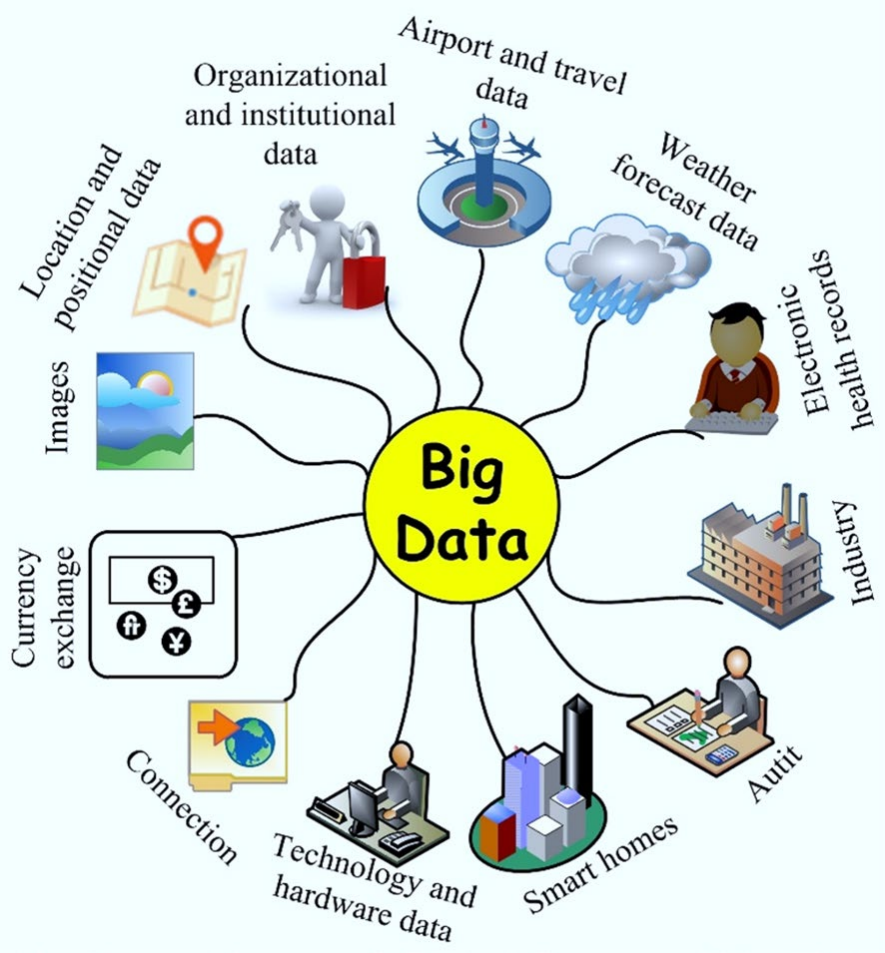
Main steps of the research protocol.

From the last few decades, we experienced an unprecedented transformation of traditional healthcare systems to digital and portable healthcare applications with the help of information systems, medical technology and other imaging resources^16^. Big data are radically changing the healthcare system by encouraging the healthcare organizations to embrace extraction of relevant information from imaginary data and other clinical records. This information will produce high throughput in terms of accurate disease diagnosing, plummeting treatment cost increase availability. In data visualization context the term ‘big data’, is firstly introduced in 1997^17^, posed an ambitious and exceptional challenge for both policy-makers and doctors with special emphasis on personalized medicine. Nonetheless, data gathering moves faster than both data analysis and data processing, emphasizing the widening gap between the rapid technological progress in data acquisition and the comparatively slow functional characterization of healthcare information. In this regard, the historical information (phonotypical and other genomic information) of an individual patient form electronic health records (EHR) are becoming of critical importance. Figure 1 represents the primary sources of big data.

Significant research work has been reported in the domains of healthcare big data analytics. To process this vast amount of information in timely manner and identify someone’s health condition based on his her is more difficult. Researchers proposed numerous applications to address this problem such as; Syed et al.^18^ proposed a machine learning-based healthcare system for providing remote healthcare services to both diseased and healthy population using big data analytics and IoT devices. Venkatesh et al.^19^ developed heart disease prediction model using big data analytics and Naïve Bayes classification technique. Kaur et al.^20^ suggested a machine learning (ML) based healthcare application for disease diagnosing and data privacy restrictions. This model works by considering different aspects like activity monitoring, granular access control and mask encryption. Some researchers presented review and survey papers to outline the recent published work in a specific directions such as Patel and Gandhi reviewed the literature for identifying the machine learning approaches proposed for healthcare big data analytics^21^. Rumbold et al.^22^ reviewed the literature for find the research work reported for diabetic diagnosing using big data analytics.

From the above discussions, it is worth mentioning that most of the researchers and industrialists gave significant attention towards the development of new computational models or surveyed the literature in a specific research direction (heart disease detection, diabetes detection, storage and security analysis etc.), but no significant research work is reported to systematically analyze the literature with different perspectives. To address this problem, this research work presents a systematic literature review (SLR) work to analyze the literature reported in healthcare big data analytics domain. This systematic analysis will not only find the gaps in the available literature but it will also suggest new directions of future research to explore.

## Research framework

**Figure 2:**
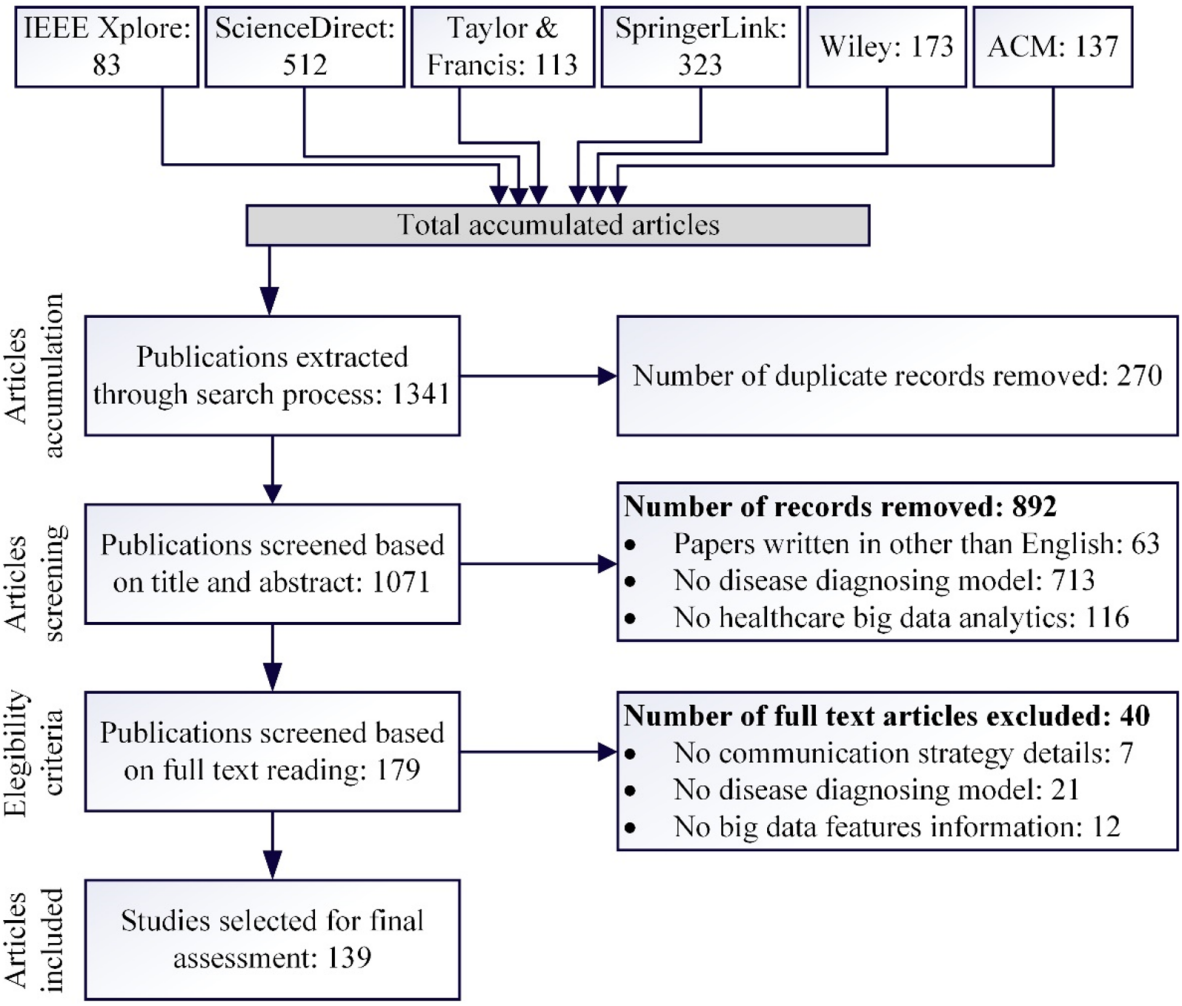
PRISMA process model for articles accumulation, screening, and final selection.

Systematic literature reviews and meta-analysis has gained significant attention and became increasingly important in healthcare domain. Clinicians, developers and researchers follow SLR studies to get updated about new knowledge reported in their fields^23,24^, and they are often followed as a starting point for preparing basic records. Granting agencies mostly requires SLR studies to ensure justification of further research^25^, and even some healthcare journals follows this direction^26^. Keeping these SLR applications in mind the proposed systematic analysis is performed following the guidelines presented by Moher et al.^27^ (PRISMA) and Kitchenham et al.^28^. This SLR work accumulates the most relevant research work from primary sources. These papers are then evaluated and analyzed to grab the best results for the selected research problem. Figure 2 represents the results after following the PRISMA guidelines. This systematic analysis are performed using the following preliminary steps:

- Identification of research questions to systematically analyze the proposed field from different perspectives.
- Selection of relevant keywords and queries to download the most relevant research articles.
- Selection of peer-reviewed online databases to download relevant research articles published in healthcare big data domain during the period ranging from 2011 – 2021.
- Perform inclusion and exclusion process based on title, abstract and the contents presented in the article to remove duplicate records.
- Assess the finalized relevant articles for identifying gaps in the available literature and suggest new research directions to explore.

### Research questions

Selecting a well-constructed research question(s) is essential for a successful review process. We formulate a set of five research questions based on the Goal Questions Metrics approach proposed by Van Solingen et al.^29^. The formulated research questions are depicted in Table 1 below.

**Table 1:** Set of Research questions.

Research questions	Description and motivation
RQ1. What are the key features adapted to integrate structured and unstructured data in healthcare big data domain?	Big data can handle a plethora of types of data including images, temporal information, EHR data, business and audit relevant data, and many other structural and non-structural data. The prime concern of this research question is to outline the available techniques proposed in the literature to handle structured and unstructured data in healthcare domains
RQ2. What are different techniques proposed to provide an easy and timely data-access interface for doctors?	Big data comprises a gigantic amount of sensitive information. It is highly secure and only authentic person can access it. Normally it takes more time in verification, data providence and in simulations to extract relevant information. And in this over-populated real-life scenario, the caretakers face more patient in less timings. To overcome this problem, how many different techniques suggested and what are the gaps in this solution to be addressed in future are the key concern of this research question
RQ3. What are different ways to improve communication between the doctor and patient?	The prime objective of this research question is, to develop an optimal communication model for both doctor and patients by enlisting the communication problems (language barrier, lack of facilities, old age or handicap personnel etc.) in the available systems. This efficient model will change the overall healthcare system
RQ4. What are different types of classification models proposed for accurate disease diagnosing using patient historical information?	This question aims to outline multiple IoT-based and machine learning-based disease diagnosing applications suggested by researchers using patient’s historical information. It also aims to discuss each model capabilities based on the information provided in the article
RQ5. What are different applications of big data analytics in healthcare domain?	The prime focus of this research question is, to extend big data analytics to new healthcare applications by outlining the current available applications of healthcare big data analytics

### Search strategy

Search strategy is the key step in any systematic research work because this is the step that ensures the most relevant article for the analysis and the assessment process. To define a well-organized search strategy a search string is developed using the formulated relevant keywords. For the accumulation of most relevant articles for a certain research problem, only keywords are not sufficient. These keywords are concatenated in different strings for searching articles in multiple online repositories^30^. Inspired from the SLR work of Achimugu et al.^31^, in software requirement domain, our search strategy consists of four main steps includes identification of keywords relevant to selected research problem, formulation of search string based on the keywords, and selection of online repositories to accumulate relevant articles to the problem selected.

#### Selection of keywords

List of keywords are defined for each research question to download all relevant articles. Some researchers defined a generic query^32^ and starts downloading articles. Although it is simple for the accumulation of articles from online database but mostly it tends to skip some most relevant articles. So, the correct option is to define keywords for each research question. In fact, it is a hectic job, but it ensures the retrieval of each relevant article from online databases regarding a certain research problem.

#### Formulation of search string

Search strings (queries) are formulated using the keywords identified from the selected research questions. The search string is tested in online databases and was modified according to retrieve each relevant articles from these databases. Inspired from the guidelines proposed by Wohlin^33^, following are the key steps undertaken to develop an optimal search string:

i. Identification of key terms from the formulated topic and research questions
ii. Selection of alternate words or synonyms for key terms
iii. Use “OR” operator for alternating words or synonyms during query formation
iv. Link all major terms with Boolean “AND” operator to validate every single keyword.

Following all these preliminary steps a generic query/search-string is developed that is depicted in Table 2. This generic query is further refined for each research question as depicted in Table 3 to retrieve each relevant article.

**Table 2:** Generalized query.

(Features OR Characteristics OR Values) AND (Big Data OR Healthcare Big Data) AND (Management OR Process OR EFFICIENT MANAGEMNT) AND (Disease Detection OR “Disease Diagnosing) AND (Patient History OR Medical Records OR Treatment Record) AND (Applications OR Uses)

**Table 3:** List of online datasets.

S. no	Online repository (s)	Permalink	Article downloading date
i	*Science Direct*	https://www.sciencedirect.com/	June 13, 2021
ii	*Springer*	https://link.springer.com/	June 15, 2021
iii	*Taylor & Francis*	https://www.tandfonline.com/	June 17, 2021
iv	*ACM*	https://dl.acm.org/	June 17, 2021
v	*IEEE Xplore*	https://ieeexplore.ieee.org/Xplore/home.jsp	June 18, 2021
vi	*Wiley online*	https://onlinelibrary.wiley.com/	June 18, 2021

#### Selection of online repositories

After identifying keywords and formulating search strings the next step is to download relevant articles specific to the interested research problem. For the accumulation of relevant articles six well-known and peer-reviewed online repositories are selected, as depicted in Table 3.

### Articles accumulation and final database development

For relevant articles accumulation and final database development we followed the guidelines suggested by Kable et al.^34^. After specifying the research questions, identifying keywords, and formulating search queries, and selecting online repositories, the next key step is to develop a relevant articles database for the analysis and assessment purposes that includes three prime steps: (1) identification of inclusion/exclusion criteria for a certain research article(s), and (2) Relevant articles database development. These steps are discussed in detail below.

#### Inclusion and exclusion criteria

After selecting online database and starts the articles downloading process, the most tedious task that the author (s) facing, is the decision about whether a certain paper should be included in the final database or not? To overcome this problem an inclusion and exclusion criteria is defined for the inclusion of a certain article in the final set of articles. Table 4 represents the inclusion and exclusion criteria followed for this systematic research work.

**Table 4:** Inclusion of exclusion criteria.

**Inclusion criteria**
1. Select papers that are only presented/written in English language
2. Select the papers that are published during the period 2011–2020
3. Include papers whose page limit size is greater than or equal to 3
4. Select only those book chapters, workshop papers or conference papers that has relevant information to our main topic
5. Select only primary research articles for the analysis purposes
**Exclusion criteria**
1. Gray papers should be excluded
2. Exclude those papers that has no relevant information regarding the selected topic
3. Elimination of duplicate research papers

A manual process is followed by the authors for the inclusion and exclusion of a certain article. These articles are evaluated based on title, abstract and information provided in the overall paper. If more than half authors agree upon the inclusion of a certain article based on these parameters (title, abstract, and contents presented in the article), then that paper was counted in the final database otherwise rejected. A total of 134 relevant primary studies are selected for the final assessment process. To ensure no skip of relevant article snowballing is applied to retrieve each relevant article.

**Figure 3:**
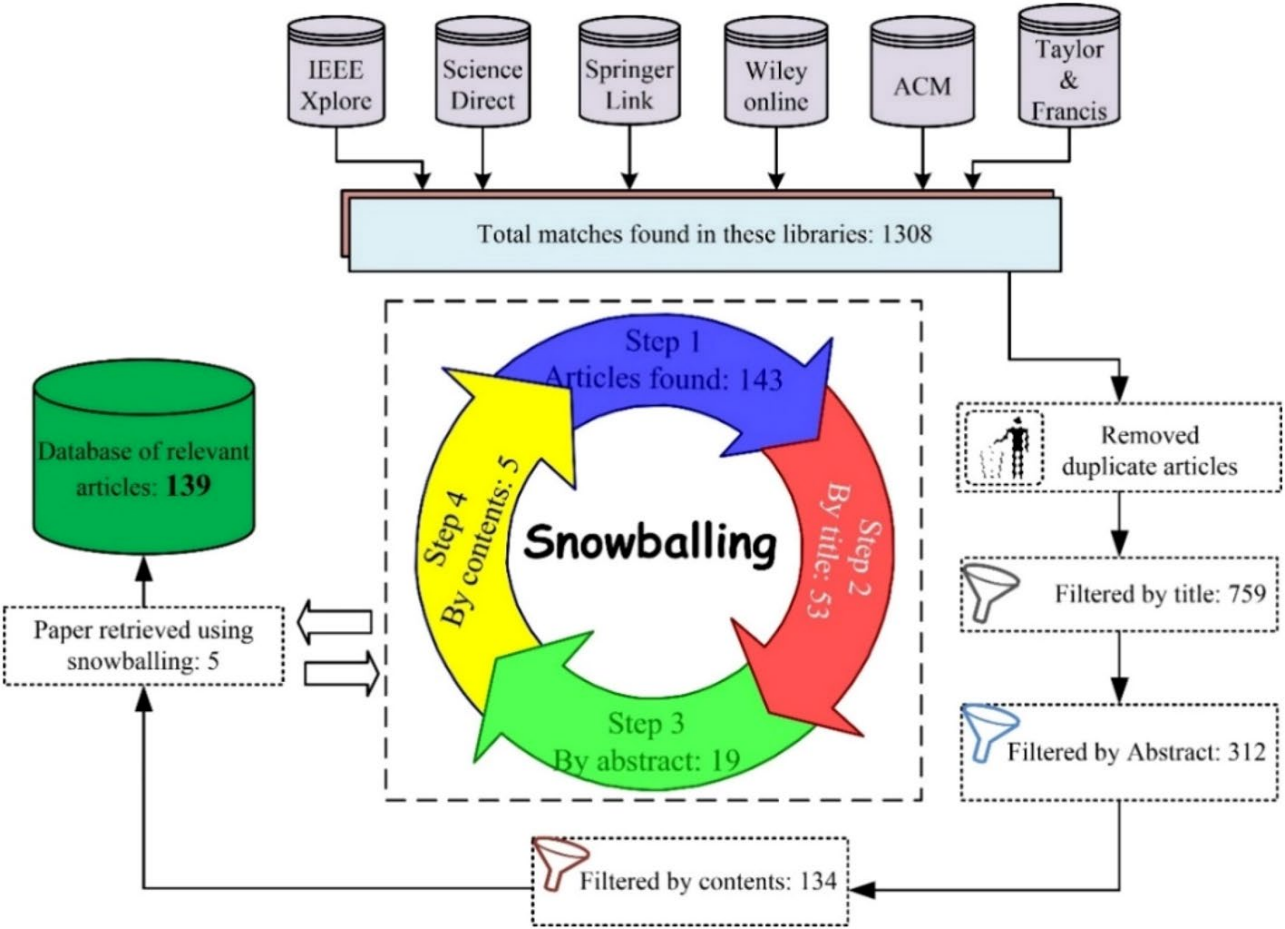
Extraction of each relevant article using snowballing.

- *Snowballing* To extract each relevant primary article snowballing is applied in the proposed research work^33^. In this systematic analysis both types of snowballing backward and forward snowballing is applied to ensure extraction of each relevant primary article. 145 relevant articles retrieved after applying snowballing process. These articles are then filtered by title and resulted for 53 relevant articles. After further processing by abstract resulted into 19 articles, and at last when filtered by contents presented in the paper resulted into only 5 relevant articles. This overall process is depicted in Fig. 3. After adding these articles to the accumulated relevant articles, a total of 139 articles added to the final database.

#### Relevant articles database development

**Figure 4:**
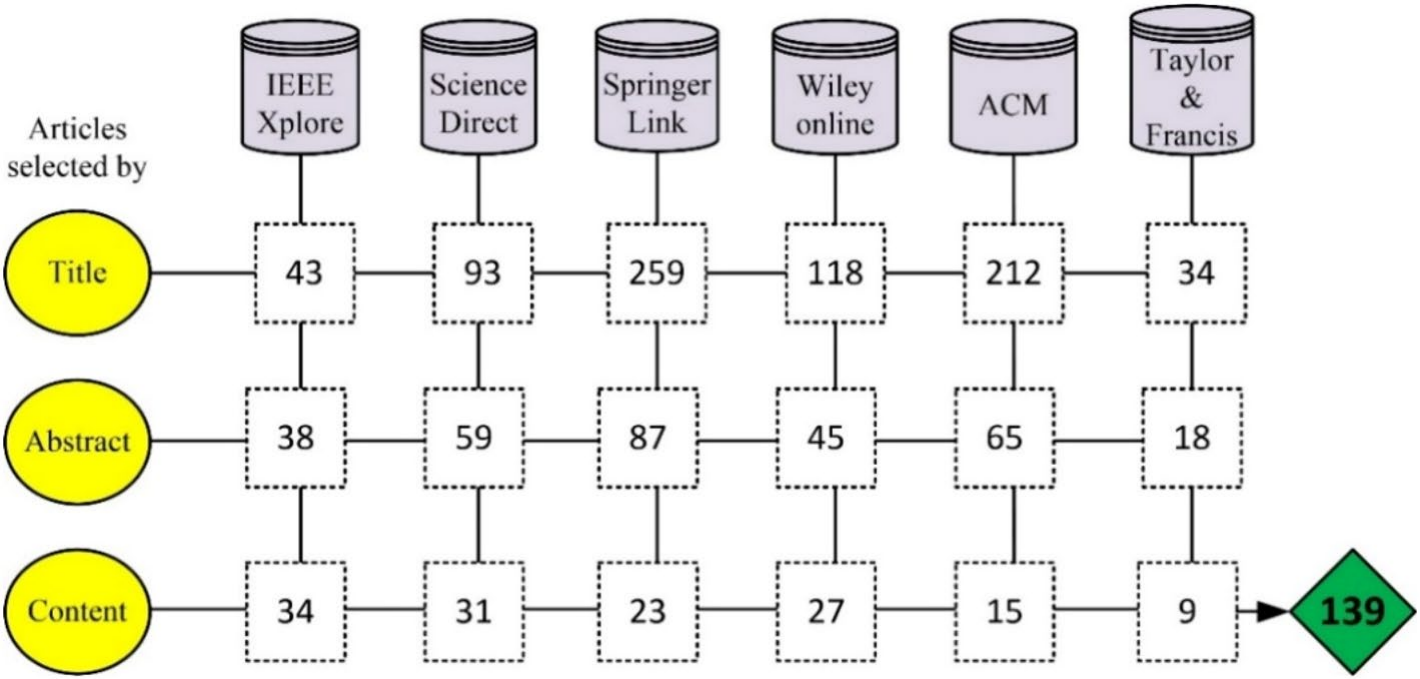
Distribution of primary studies.

After accumulating each primary article reported in the proposed field, a database of relevant articles is developed for the assessment and analysis work, to find the current available trends in healthcare big data analytical domain and investigate the gaps in these research articles to open new gates for future research work. A total of 139 relevant articles are added to the final database. The overall contribution of the selected online repositories in the relevant articles database development is depicted in Fig. 4.

From Fig. 4, it is concluded that IEEE Xplore and Science Direct contributing the more that reflects the interest of research community to present their work with.

### Articles accumulation and final database development

**Figure 5:**
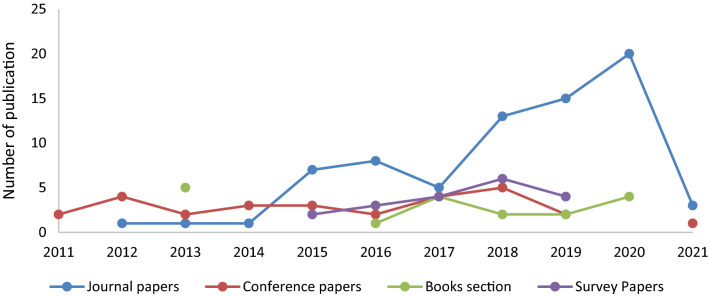
Evolution of final database by type of article and year.

After developing a database of relevant articles, it is evaluated using different parameters like type of article (conference proceedings, journal article, book chapter etc.), publication year, and contribution of individual library. Figure 5 represents the information regarding the total contribution of articles by type in the final database.

**Figure 6:**
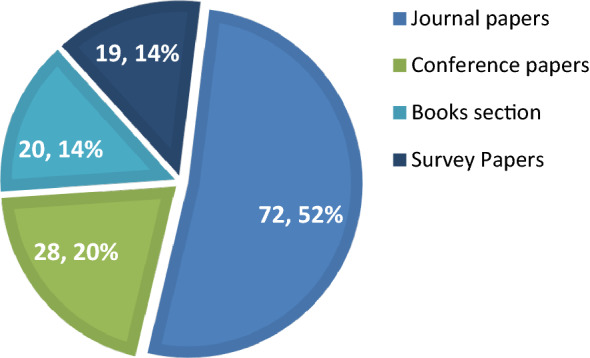
Percentage contribution by type of paper.

Figure 5 concludes that the researchers paid significant attention towards the development of new healthcare systems instead of finding the gaps in the available systems and develop enhanced solutions accordingly. This enhanced solution can accurately identify and diagnose a certain disease based on patient’s historical medical information. A small amount of work is reported using review articles, survey papers, but no systematic mechanism is followed to analyse the work in specific range of years followed by a set of research questions. The same problem can also be seen from Fig. 6 where highest percentage contribution is shown more comparative to book sections, conference papers etc.

**Figure 7:**
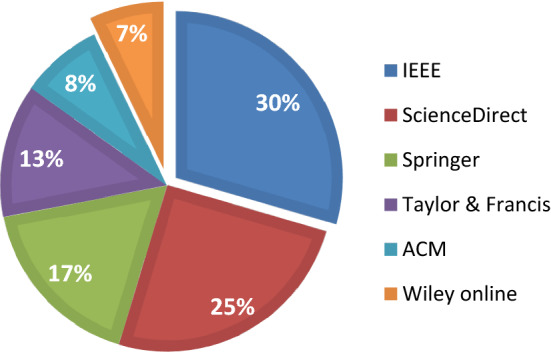
Percentage contribution of each library.

Figure 7 depicts the percentage contribution of each library in the proposed assessment work.

**Figure 8:**
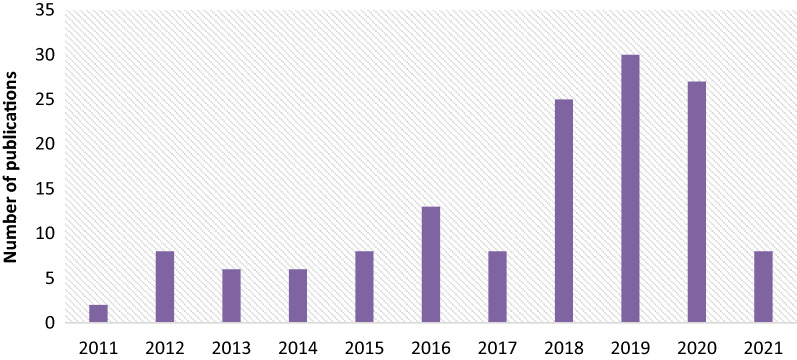
Annual distribution of articles.

Figure 8 represents the annual distribution of articles selected for the analysis and assessment purposes. Form Fig. 8 it is evident, that with passage of time number of articles increases, and that shows the maturity and interest of the researchers in this specific domain.

**Figure 9:**
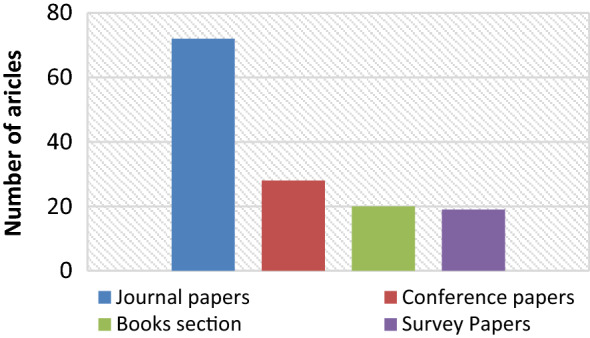
Evolution of database by number of articles by type.

From Fig. 8, it is concluded that IEEE Xplore contributing the more in the final database of relevant articles that shows the trend of researchers to present healthcare relevant works in the IEEE journals. Figure 9 represents the total number of journal articles, survey papers, conference papers, and book sections in the selected relevant articles database.

From Fig. 9 it is concluded that significant attention is given towards the development of new healthcare models. This shows the maturity of the proposed field. Dealing with such a mature field and extracting useful information is hectic job for the researchers. A systematic analysis of this research field is needed to provide an overview of the work reported during a specific range of years. This analysis will not only save precious time of the researchers, but it will also open gates for the future research work in this field.

Table 5 represents the annual contribution of studies in the final relevant database.

**Table 5:** Inclusion or exclusion criteria.

Year	Paper ids
2011	^35,36^
2012	^34,37–44^
2013	^3,45–49^
2014	^16,50–54^
2015	^4,55–65^
2016	^66–83^
2017	^11,84–94^
2018	^9,10,20,21,95–123^
2019	^7,8,18,19,22,124–152^
2020	^15,153–183^
2021	^167,184–190^

**Figure 10:**
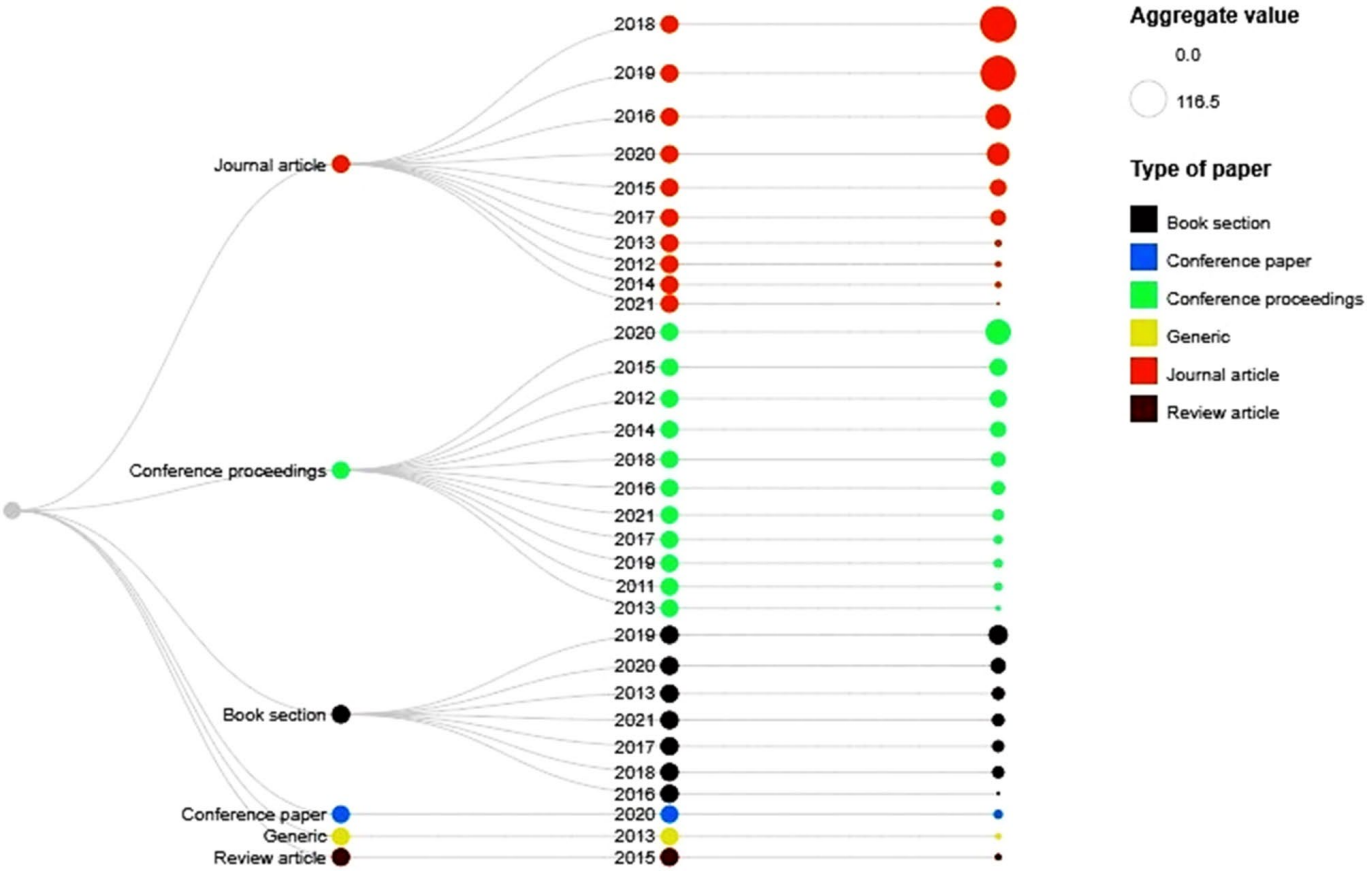
Evolution of final database.

Overall information regarding type of paper, publication year and number of records is depicted in Fig. 10 below.

## Quality assesment

After executing exclusion and inclusion process, all the relevant articles in the database are manually assessed by authors to check the relevancy of each article with the selected research problem. A quality criterion is defined to check every research article against the formulated research questions. This quality criteria is defined in Table 6.

**Table 6:** Quality criteria.

S.no	Quality criteria
*QC1*	What are the key features selected for the optimum organization of both structural and non-structural data in healthcare big data domain?
*QC2*	Whether an algorithm proposed for the development of an optimal and timely data access interface for doctors to treat patients?
*QC3*	Whether any technique proposed to develop an accurate communication model for both patient and doctor?
*QC4*	Using patient historical information, whether a diagnosing model suggested for the accurate detection of a certain type of disease?
*QC5*	Whether healthcare big data analytics is proposed for certain type of application?

**Figure 11:**
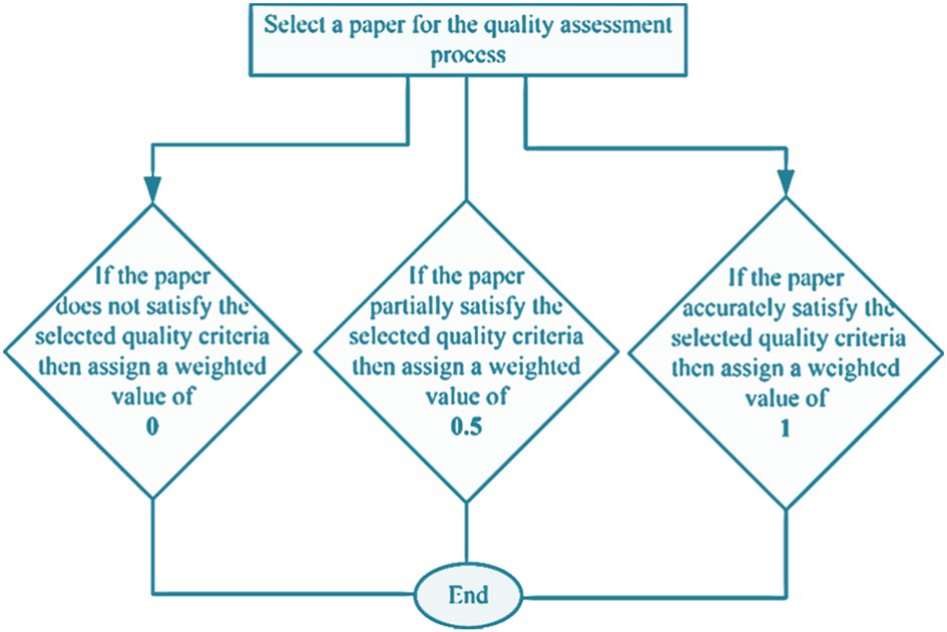
Quality criteria for the proposed SLR work.

Weighted values are assigned against each quality criteria to check the relevancy of an article with a certain research question. These weighted values and description is depicted in Fig. 11.

**Figure 12:**
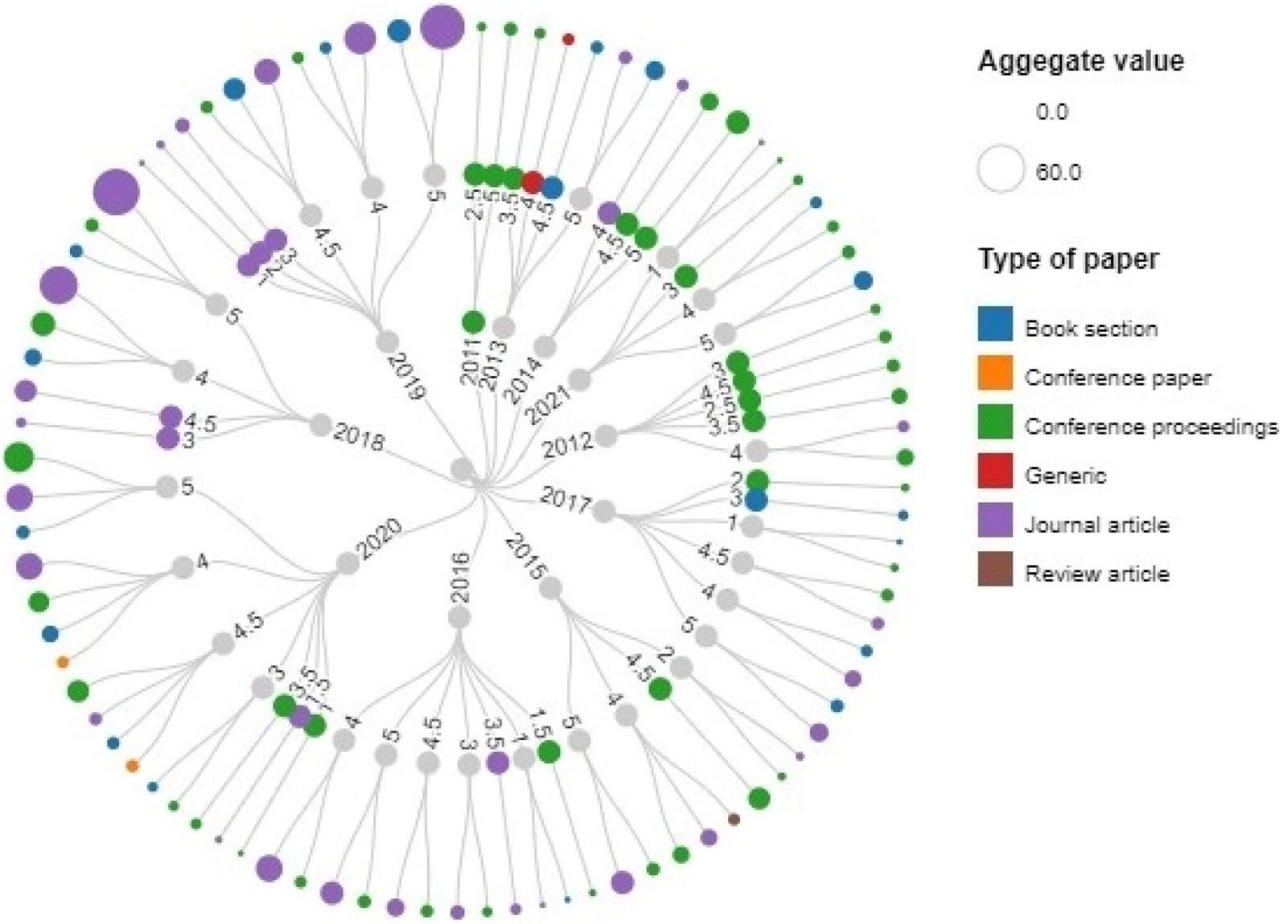
Quality assessment process.

After the assessment process, the relevancy of each article is decided based on its aggregated weighting score. If the score is greater than 3 it represents the most relevancy of an article to the selected research topic. Figure 12 represents the aggregate score values of each article based on the defined quality assessment criteria.

## Results and discussion

After executing the quality assessment work, the next key step of an SLR work is, to analyse all the relevant article to identify different techniques proposed for efficient communication between patient and practitioner, accurate feature extraction from healthcare big data and implement it in practical use.

This section of the paper performs a descriptive analysis of each article based on five research questions. In this systematic review process, a total of 139 research articles published during the period ranging from 2011 to 2021.

### Healthcare big data

**Figure 13:**
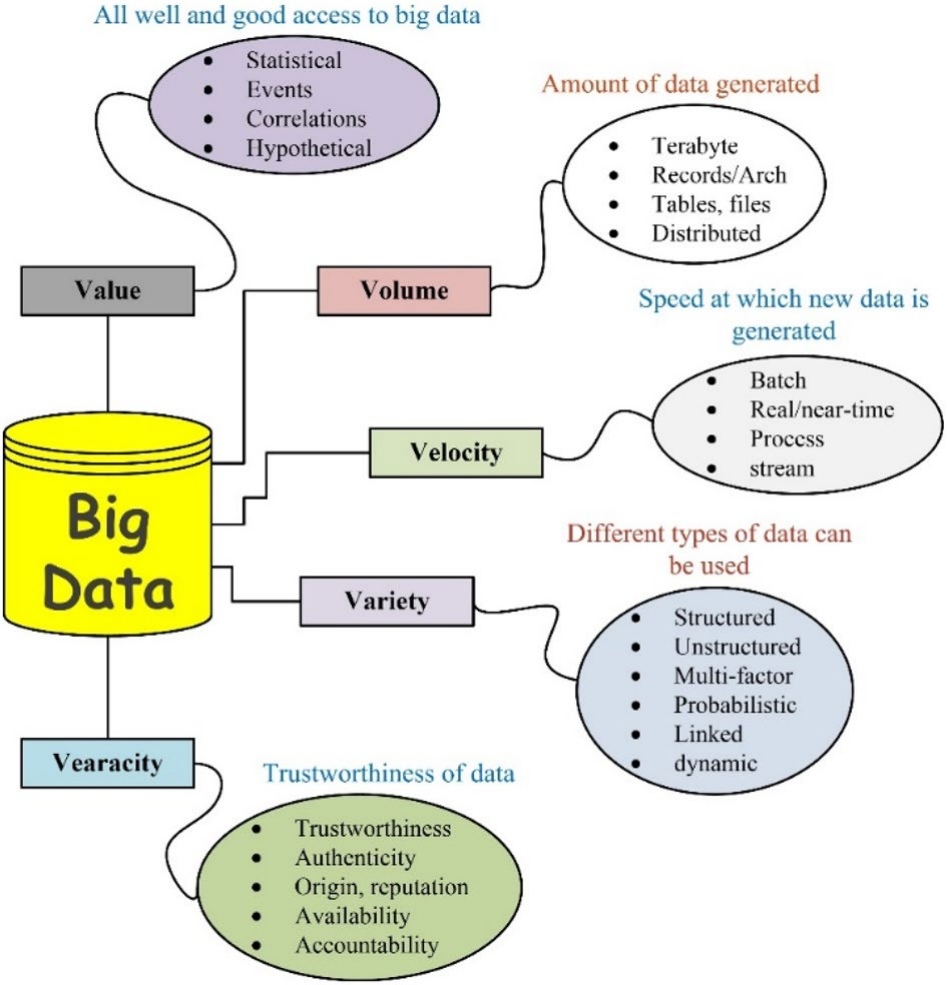
Big Data 5Vs^15^.

The researcher and data analysts suggested no contextual name for “big data” in healthcare, but for implementation and interpretation purposes they divided it into 5 V architecture. Figure 13 depicts a 5 V architecture of big data.

The exponential increase in IoT-based smart devices and information systems resulted a plethora of information in healthcare domain. This information increases exponentially on daily basis. These smart IoT based healthcare devices produces a huge of data. An alternated term “Big Data” is selected for this gigantic amount of data. This is the data for which scale, diversity, and complexities require innovative structure, variables, design, and analytics for efficient utilization and management, accurate data extraction and visualization, and to grab hidden stored information regarding a specific problem of interest. Main idea behind the implementation of healthcare big data analytics is to retrieve enriched information from huge amount of data using different machine leering and data mining techniques^191^. These techniques help in improving quality of care, reducing cost of care, and helps the practitioners to suggest medicines based on clinical historical information.

### RQ1. What are the key features adapted to integrate the structured and unstructured data in healthcare big data domain?

Big data comprises a huge amount of data to be processed, especially a plethora of types of data to process and extract enriched information regarding a problem of interest. Several features are assessed and analyzed especially in healthcare domain, to integrate both structural and non-structural data. Multiple researchers analyzed semantic based big data features for big data integration purposes while some researchers proposed behavior and structural based features for patient monitoring and activity management purposes^151,192^. While some performed real-time analysis using a group of people for data integrating and clustering purposes. Table 7 enlists the research work published for the structural and non-structural data integration purposes.

**Table 7:** List of key features used for data integration purposes.

S. no	Citation	Key features	Description
1	^42^	Structural features	Using the CT scan images and healthcare big data analytics, this paper identified the normal and abnormal brain structure condition with the help of structural based features
2	^193^	Behaviour-based features	This article summarizes the action-based (exemplar-based) features to improve the capabilities of nursing staff in some unwanted and emergency situations. It mainly focuses on the exploration of exemplars from nursing informatics research, to develop a smart mechanism that is feasible for nursing staff to participate in the big data revolution
3	^60,119^	Semantic based features	Around the world, a standard format of documents relevant to healthcare are suggested for the healthcare assessment and analysis process but the companies and the researchers face a big barrier during its comprehension and timely processing. To address this problem Hadoop based semantic transformation model is presented in this research work by using semantic based features with the help of CAD architecture
4	^64^	Heterogeneous data features	For monitoring quality of life of an individual patient several features are considered for the analysis and assessment processes. These features include non-textual information, heterogeneous data spaces, organ and organism scale, and specified analytics for identifying “physiological envelope” for a patient routine assessment purpose
5	^67,83^	Activity-based features	Social networking is an alternate source for retrieving information regarding daily healthcare activities. Social media facilitates the user by proving an easy access environment without any long time wait in queue or interruption compared to the traditional health pooling stations, where both younger and elder people wait for long time for their turn to poll. A Facebook application is proposed in this research work, where more than 1400 employees selected to accumulate patient routine-based activity information. This data can be collected in relatively small interval of time compared to the traditional arguing system without any promotional action
6	^105^	5 V features	5 V features are outlined in this book chapter to integrate both structural and non-structural data in healthcare big data domain. These features include volume, velocity, veracity, value, and variety-based features. It also consists other features such as: vulnerability and complexity
7	^44^	Real-Time analysis-based features	This research article presents a real time scenario for feature accumulation and disease diagnosing purposes. In this case study seven patient were contributed for the prediction of seizure disease using support vector machine
8	^36^	Integrated features	With rapid improvement and ever-increasing data in healthcare domain make it more complex to retrieve data regarding specific research oriented and disease diagnosing problem. To address this critical problem Tian^36^ presented the concept of integrated features to integrate CT scan images in big data domain for efficient retrieval and disease diagnosing purposes
9	^194^	Functional features	This systematic review work has outlined the use of cognitive computing and functional features reported in healthcare, cybersecurity, and big data

After analysing the available literature in Table 8, it was concluded that mostly semantic based, structure-based, and real-time activity-based features are considered for the information extraction and organization purposes. If we consider geometric based feature and adapt clustering mechanism for data organization purposes, then this will not only integrate both structural and non-structural data efficiently, but it will improve the simulation capabilities of different applications.

**Table 8:** List of techniques proposed for easy and time data-access interface.

No	Citation	Technique	Description
1	^56^	Network foresight approach	To provide an easy and timely data-access interface this research work used networked foresight approach for the prediction of accurate medicine using current and past clinical historical information. This approach provides an easier interface for the patients and doctors to communicate and exchange information regarding their health status
2	^8–11,19,57,63,73,78,82,86,92,95,97,111–113,116,120,123–126,128,129,131–133,135,137,139–141,144–147,149–154,156,157^	Big data analytics for timely data-access purposes	These survey papers presents applications of big data analytics in term of facilities like presenting an easier and timely-access mechanism for both patients and practitioners
3	^70,197^	Pig Latin Script programming tool	Pig Latin Script is applied to analyse the healthcare datasets against different queries, to enhance health facilities in India by assessing healthcare big data. It also provides an optimum and timely data-access mechanism for both patient and practitioner
4	^83^	Social media-based applications	This research article performed data analysis approach on data retrieved from Facebook walls. In this analysis process 153 public organization were contributed to accumulate information regarding patient activities and performance using unsupervised classification models
5	^91^	IoT-based systems	This paper outlined different IoT-based applications, cloud computing-based application, embedded systems, and other smart applications proposed for smart healthcare purposes at door step (without visiting the practitioners on daily basis)
6	^94,109^	eHealth applications	The overpopulation and complexities in medical and treatment makes the things complex for the patients to access their basic health providers on time. And this situation becomes more critical in 3rd world countries. To tackle this issue a smart phone-based eHealth application is developed to provide clinical facilities at homes. It also provides an easy interface for both doctors and patients to communicate easily and in timely fashion
7	^100^	Formal methods	Overpopulation, treatment errors, and overtreatment results in developing a disease burden situation and health disparities by combining with noncontagious disease population. Formal methods are applied to develop an optimum mechanism with zero defects for timely and accurate disease diagnosing and treatment purposes
8	^104^	Medicine suggestion interface	This paper presents an automatic medicine suggestion interface by extracting information from the patient’s clinical records. This model not only enhanced the efficiency of doctors to check multiple patients in a timely fashion but also enhance his/her capabilities to advice accurate medicine for a certain type of disease
9	^106^	ML techniques	This paper surveyed the literature by finding the gaps in the available mHealth applications and developed a new mHealth2.0 application. This advanced version is developed using machine learning (ML) techniques to simulate big data in healthcare. This application provides enhance quality of care services to the patients
10	^22,121^	Statistical analysis-based models	This research work has developed a mathematical model for diabetic’s analysis using big data. This analytical model helped the doctors by providing an easy simulation interface to process patient’s clinical records in a short time to monitor patient’s current diabetic situation remotely

### RQ2. What are different techniques proposed to provide an easy and timely data-access interface for doctors?

Digital transformation of healthcare systems by using of information system, medical technology, handheld and smart wearable devices has posed many challenges for both the researchers and caretakers in the form of storage, dropping the cost of care and processing time (to extract relevant information for refining quality of care and reduce waste and error rates). Prime goal of healthcare big data analytics is, to process this vast amount of data using machine learning and other processing models to extract certain problem relevant information and use it for human well beings^195^. Several supervised and unsupervised classification techniques are followed for the said purposes. ML-based architectures and big data analytical techniques are integrated in healthcare domain for efficient information retrieval and exchange purposes, risk analysis, optimum decision-support system in clinics, and suggesting precise medicines using genomic information^196^. Table 8 represent the literature reported for the providence of an easy and timely data-access interface for the practitioners.

### RQ3. What are different ways to improve communication between the doctor and patient?

Healthcare around the world is under high pressure due to limiting financial resources, over-population, and disease burden. In this modern technological age, the healthcare paradigm is shifting from traditional, one-size-fits-all approach to a focus on personalized individual care ^1^. Additionally, the healthcare data is varying both in type and amount. The healthcare providers are not only dealing with patient’s historical, physical and namely information, but they also deal with imaging information, labs, and other digital and analogue information consists of ECG, MRI etc. This data is voluminous, varying in type and formats, and of differing structure. These are the capabilities of Big Data to handle not only different types of and forms of data, but can handle 5 V structure including volume, variety, value, veracity, and velocity. Thus, the doctors facing an increasing burden of rising patient numbers coupled with progressively less time to spend with each patient. In other words, we are dealing with more patients, more data, and less time.

Different techniques are proposed in the literature to provide an easy and timely communication interface for both doctors and patients. Table 9 depicts different information exchange tools/techniques reported in the literature.

**Table 9:** List of techniques proposed for efficient communication and information exchange.

No	Citation	Technique	Description
1	^59^	Analytical framework	This research article proposed an analytical framework using big data analytics for the identification of Lupus disease. This model presents an easy interface for the doctors to diagnose Lupus disease on patient’s clinical records. In this smart framework, the patients upload their EHR and the doctor performs the Lupus disease diagnosing
2	^69^	Bio Inspired ICT interface	In this modern technological age, the human health and life-loss will be purely dependant on data and its optimum organization. But organizing a huge amount of data generated on daily basis from industries, smart and portable IoT healthcare devices, and many others is a hectic job. This paper presents a Bio inspired ICT interface to present an intelligent healthcare application to both doctors and the patient. This application makes the doctor and patient convenient to exchange the information easily and in timely fashion
3	^76^	Cloud-based architecture	An optimal data exchange mechanism is developed in this research article by presenting the concepts of cloud computing. Totally different from the traditional database system that are full of anomalies. This cloud-based architecture makes the doctors convenient to store an individual patient’s clinical records in a standard format that are easy to extract, update, and process for future purposes
4	^80,115^	Hybrid model	This paper embeds multiple integrated applications such as IoT-based systems, sensors and arrays of sensors, big data analytics for emerging technologies. This hybrid model ultimately provides a smart application in low cost, low data storage requirements, with high communication capabilities in between the patient and practioners
5	^84,85,107,118^	Secure communication framework for doctor and patient	On one side the available of smart IoT-based applications made our life easier by presenting an easy access interface. But on other side it threatens our life by giving an opportunity to hackers to steal our information from distant offices. Keeping these drawbacks in mind this research work has reviewed different techniques proposed for efficient and secure communication in between patient and doctor
6	^87^	SOA-FOG based architecture	A three-tier architecture is developed for healthcare big data management using Fog computing (SOA-FOG architecture). It displays security information in fog layer, client layer, and in cloud layer. This framework presents a secured communication framework between the doctor and the patients and can address the man-in-the-middle network attack problems
7	^101^	Data mining techniques	Smart clinical intelligence model is proposed in this research article using data mining techniques. This model aims to assist the doctors to remotely process the clinical health records of a particular patient and validate his health conditions
8	^198^	mHealth 4.0	This paper surveyed different industry 4.0 applications by identifying the gaps in the available mHealth applications and suggested a new enhanced healthcare mobile smart application (mHealth 4.0). This advanced version of the mHealth application provides an easy communication facility for doctor and patient where they can exchange information easily
9	^20^	Machine learning based model	This paper has developed a hybrid ML-based model using mask encryption, monitoring patient activities, granularly access control, real time data classification techniques. This hybrid healthcare technique facilitates both doctors by presenting a more accurate disease diagnosing model
10	^199^	CrowdHEALTH	A real-time communicating framework is developed for practitioners and doctors using healthcare big data analytics and Holistic Health Records (HHR) in the context of the CrowdHEALTH. Big data analytics is executed on the database to retrieve stored records (i.e. HHRs) for clinicians and doctors to advice medications

### RQ4. What are different types of classification models proposed for accurate disease diagnosing using patient historical information?

**Figure 14:**
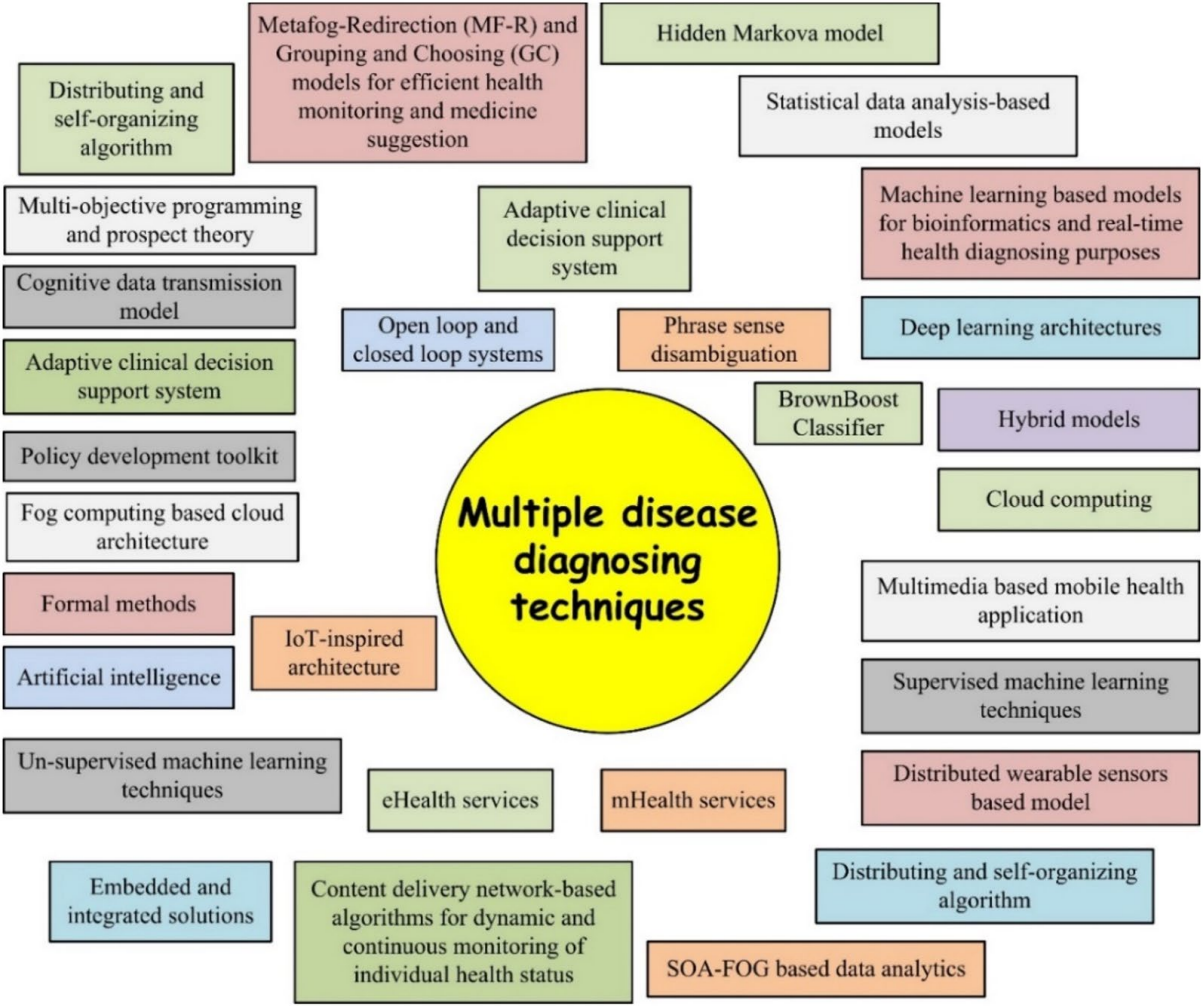
Multiple disease diagnosing techniques proposed in the literature.

This research question aims to outline different disease diagnosing models proposed in the literature using healthcare big data. Around the world diverse approaches are proposed by researchers for healthcare big data analysis to ensure accurate disease diagnosing capabilities, provide healthcare facilities at doorstep, development of eHealth and mHealth applications, and many others. Multiple statistical and ML-based approaches proposed for accurate diagnosing purposes. Figure 14 represents multiple techniques proposed for automatic disease diagnosing purposes using healthcare big data domain.

All these techniques perform the diagnosing process using semantic-based features or structural based features. But no attention is given towards geometric feature extraction techniques that are prominent in extracting enriched information from data and results in high identification rates. Also, no advanced hybrid neural network and shallow architectures are proposed for the automatic diagnosing purposes. Keeping these gaps in mind, an optimum eHealth application can be developed by applying these hybrid techniques.

### RQ5. What are different applications of big data analytics in healthcare domain?

Big data analytics has revolutionized our lives by presenting many state of the art applications in various domains ranging from eHealth to mHealth, weather forecasting to climate changes, traffic management to object detection, and many others. This research question mainly focusing on enlisting different applications of big data analytics in Table 10.

**Table 10:** Applications of big data analytics in healthcare domain.

S. no	Citations	Architectures	Description
1	^193^	Proper treatment	Healthcare big data analytics has enhanced the capabilities of nursing staff by practicing data science and analysis for accurate treatment using patients’ clinical-relevant historical information
2	^59^	Lupus disease diagnosing	This research work developed lupus disease diagnosing system with the help of big data analytics
3	^64^	Silico medicine solutions	Integrating VPH technologies with healthcare big data analytics a robust and effective solution is developed in this research work that provides a silico medicine solution for individuals
4	^200^	Real-time health analysis	A smart real-time eHealth application is presented in this paper by embedding Amazon and Microsoft Azure services to solve health issues in a limited time
6	^71^	Alzheimer’s disease detection	A statistical data analysis mechanism is proposed in this research work for the identification of Alzheimer’s disease using Chronic nervous system and healthcare big data analytics
7	^88^	health risk assessment	Multiple eHealth applications are overviewed in this research work to develop an optimal technique for patient’s health stratification and risk assessment purposes
9	^93^	eHearing loss application	An embedded solution is proposed in this research work for hearing loss (HL) patients. This application uses holistic management scheme to the HL-patients by facilitating public health services. This integrated solution also keeps the users away from this type of diseases in future
11	^110,201^	High storage capabilities	Big data has a large amount of data to be processed, particularly a plethora of types of data to process and extract enriched information regarding a problem of interest. Furthermore, storing this vast amount of data is another big challenge for the research community. Apache Pig and Apache HBase are the repository facilitates the research community to store this huge amount of information easily and provides an easy access interface for the retrieval purposes
12	^114^	Health status detection	This research work presents a machine learning-based model for checking the patient’s health status. Apache Spark-based cloud computing and data processing engine is deployed to process tweets (related to a specific patient’s health status)
13	^121^	Hadoop framework	Using healthcare big data analytics, this study work proposes a statistical diagnosing mechanism for diabetic analysis. Hadoop framework is used for the validation and implementation purposes
14	^128,131,142^	Indoor treatment system	In this over-populous and disease burden environment accessing hospitals and doctors for basic health purposes is more difficult. But this problem become more severe for old age patients. To address this problem, these research articles propose an IoT-based smart eHelath application that facilitates aged personnel at doorstep
15	^7^	Intelligent hospital appointment system	A smart hospital appointment system was developed in this research article by using the concept of first come-first serve (FIFO) concept. This model not only vanished the concept of traditional doctor-patient appointment system but also helps in fixing the appointment with an expert doctor using healthcare data bank
16	^139^	Digital HIV/AIDS detection system	This research article presents an electronic HIV/AIDS detection system using healthcare big data analytics. Then a regional based monitoring system is developed to monitor these patients. This model not only helps in identifying the HIV/AIDS infected patient in a specific region, but it also helps the practitioners, researchers, and government agencies to perform proper treatment of these patients
17	^145^	Parkinson disease diagnosing model	This study has developed a sensor-based robot model for Parkinson disease diagnosing purposes. Sensors are integrated in the infected patient’s body. Based on the sensed data the robot moves with the patient and accumulate data. This accumulated data was then provided to artificial neural network for classification and recognition of Parkinson disease

## Limitations

This article has a number of limitations. Some of these limitations are listed below.

- For this systematic analysis articles are only accumulated from six different peer-reviewed libraries (ACM, SpringerLink, Taylor & Francis, Science Direct = IEEE Xplore, and Wiley online library), but there exist a number of multi-disciplinary databases for articles accumulation purposes.
- This systematic analysis covers a specific range of years (2011 –2021), while a number of articles are reporting on daily basis.
- Articles are accumulated from online libraries using search queries, so if a paper has no matching words to the query, then it was skipped during search process.
- Google Scholar is skipped during the articles accumulation phase to shorten the searching time. Also, it gives access to both peer-reviewed and non-peer-reviewed journals and we only focused on peer-reviewed journals for the relevant articles.
- Being a systematic literature work it can be broadened to grab the knowledge about other varying topics such as healthcare data commercialization, health sociology etc.

Besides these limitations we hope that this systematic research work will be an inspiration for future research in the recommended fields and will open gates for both industrialists and policymakers.

## Conclusion and future work

In this research article, the existing research reported during 2011 to 2021 is thoroughly analysed for the efforts made by researchers to help caretakers and clinicians to make authentic decisions in disease diagnosing and suggest medicines accordingly. Based on the research problem and underlying requirements, the researchers proposed several feature extraction, identification, and remote communication frameworks to develop doctor and patient communication in a timely fashion. These real-time or nearer to real-time applications mostly use big data analytics and computational devices. This research work identified several key features and optimum management designs proposed in healthcare big data analytical domain to achieve effective outcomes in disease diagnosing. The results of this systematic work suggests that advanced hybrid machine learning-based models and cloud computing application should be adapted to reduce treatment cost, simulation time, and achieve improved quality of care. The findings of this research work will not only help the policymakers to encourage the researchers and practitioners to develop advanced disease diagnosing models, but it will also assist in presenting an improved quality of treatment mechanism for patients.

Advanced hybrid machine learning architectures for cognitive computing are considered as the future toolbox for the data-driven analysis of healthcare big data. Also, geometric-based features must be considered for feature extraction purposes instead of semantic and structural-based features. These geometric-based feature extraction techniques will not only reduce the simulation time, but it will also improve the identification and disease diagnosing capabilities of smart health devices. Additionally, these features can help in accurate identification of Alzheimer, tumours in PET or MRI images using upgraded machine learning and big data analytics. Cluster-based mechanism should be considered for data organization purposes to improve big data timely-access and easy-management capabilities. Promoting research in these areas will be crucial for future innovation in healthcare domain.

## Data Availability

The data used and/or analyzed during the current study available from the corresponding author on reasonable request.
